# Development and predictors of bipolar disorder in children and adolescents with depressive disorders: a systematic review, meta-analysis, and meta-regression

**DOI:** 10.1192/j.eurpsy.2024.1814

**Published:** 2025-01-08

**Authors:** Gonzalo Salazar de Pablo, Violeta Perez-Rodriguez, Javier de Otazu Olivares, Javier Camacho-Rubio, Aditya Sharma, Ana Catalán, Josefien Breedvelt, Claudia Aymerich, Mihai Pop, Carmen Moreno, Ian Kelleher, Jane Anderson, Paolo Fusar-Poli, Christoph U Correll, Allan H. Young

**Affiliations:** 1 Department of Child and Adolescent Psychiatry, Institute of Psychiatry, Psychology & Neuroscience, King’s College London, London, UK; 2 Child and Adolescent Mental Health Services, South London and Maudsley NHS Foundation Trust, London, UK; 3 Department of Child and Adolescent Psychiatry, Institute of Psychiatry and Mental Health, Hospital General Universitario Gregorio Marañón, IiSGM, CIBERSAM, ISCIII, School of Medicine, Universidad Complutense, Madrid, Spain; 4 School of Medicine, Universidad Nacional Pedro Henriquez Ureña, Santo Domingo, Dominican Republic; 5 Academic Psychiatry, Translational and Clinical Research Institute, Newcastle University, Newcastle upon Tyne, UK; 6 National Specialist Adolescent Mood Disorders Service (NSAMS), Cumbria, Northumberland, Tyne and Wear NHS Foundation Trust, Newcastle upon Tyne, UK; 7 Department of Psychosis Studies, Institute of Psychiatry, Psychology & Neuroscience, King’s College London, London, UK; 8 Psychiatry Department, Biocruces Bizkaia Health Research Institute, OSI Bilbao-Basurto. Facultad de Medicina y Odontología, University of the Basque Country UPV/EHU. Centro de Investigación en Red de Salud Mental (CIBERSAM), Instituto de Salud Carlos III. Barakaldo, Bizkaia, Spain; 9 East London NHS Foundation Trust, London, UK; 10 Centre for Clinical Brain Sciences, University of Edinburgh, Edinburgh, UK; 11 School of Medicine, University College Dublin, Dublin, Ireland; 12 Faculty of Medicine, University of Oulu, Oulu, Finland; 13 St John of God Hospitaller Services Group, Stillorgan, Ireland; 14 Department of Brain and Behavioral Sciences, University of Pavia, Pavia, Italy; 15 OASIS Service, South London and Maudsley NHS Foundation Trust, London, UK; 16 Maudsley Biomedical Research Centre, National Institute for Health Research, South London and Maudsley NHS Foundation Trust, London, UK; 17 Department of Psychiatry, The Zucker Hillside Hospital, Northwell Health, Glen Oaks, NY, USA; 18 Department of Psychiatry and Molecular Medicine, Zucker School of Medicine at Hofstra/Northwell, Hempstead, NY, USA; 19 Center for Psychiatric Neuroscience, The Feinstein Institutes for Medical Research, Manhasset, NY, USA; 20 Department of Child and Adolescent Psychiatry, Charité Universitätsmedizin, Berlin, Germany; 21 Centre for Affective Disorders, Institute of Psychiatry, Psychology & Neuroscience, King’s College London, London, UK; 22 South London and Maudsley NHS Foundation Trust, Bethlem Royal Hospital, Beckenham, UK

**Keywords:** adolescents, bipolar disorder, children, depressive disorders, meta-analysis, prevention

## Abstract

**Background:**

Estimating the risk of developing bipolar disorder (BD) in children and adolescents (C&A) with depressive disorders is important to optimize prevention and early intervention efforts. We aimed to quantitatively examine the risk of developing BD from depressive disorders and identify factors which moderate this development.

**Methods:**

In this systematic review and meta-analysis (PROSPERO:CRD42023431301), PubMed and Web-of-Science databases were searched for longitudinal studies reporting the percentage of C&A with ICD/DSM-defined depressive disorders who developed BD during follow-up. Data extraction, random-effects meta-analysis, between-study heterogeneity analysis, quality assessment, sub-group analyses, and meta-regressions were conducted.

**Results:**

Thirty-nine studies were included, including 72,371 individuals (mean age=13.9 years, 57.1% females); 14.7% of C&A with a depressive disorder developed BD after 20.4–288 months: 9.5% developed BD-I (95% CI=4.7 to 18.1); 7.7% developed BD-II (95% CI=3.2% to 17.3%); 19.8% (95% CI=9.9% to 35.6%) of C&A admitted into the hospital with a depressive disorder developed BD. Studies using the DSM (21.6%, 95% CI=20.2% to 23.1%) and studies evaluating C&A with a major depressive disorder only (19.8%, 95% CI=16.8% to 23.1%) found higher rates of development of BD. Younger age at baseline, a history of hospitalization and recruitment from specialized clinics were associated with an increased risk of developing BD at follow-up. Quality of included studies was good in 76.9% of studies.

**Conclusions:**

There is a substantial risk of developing BD in C&A with depressive disorders. This is particularly the case for C&A with MDD, DSM-diagnosed depressive disorders, and C&A admitted into the hospital. Research exploring additional predictors and preventive interventions is crucial.

## Introduction

Bipolar disorder (BD) is a chronic and debilitating disorder [1] characterized by fluctuations in mood states and energy [2]. BD significantly affects psychosocial functioning and quality of life [3, 4]. BD is also the second most common mental disorder for the effect on ‘days out of role’ functioning in young individuals [5]. Life expectancy is reduced by approximately 12–14 years in people with BD [6].

Early diagnosis and treatment are associated with a more favorable prognosis in BD. A large-scale meta-analysis of epidemiological studies including youth and adult studies estimated that the global peak age at onset of BD is 19.5 years [7]. Despite such an early onset, diagnosis and optimal treatment are often delayed by a mean of approximately 9 years following an initial depressive episode [6].

Children and adolescents (C&A) often present with a depressive episode before developing an episode of mania [8]. After the initiation of antidepressant medication, manic episodes are estimated to occur in 3–10% of C&A with unipolar depression [9].

Risk factors for BD include the presence of depressive disorders, anxiety disorders, conduct disorder, and attention-deficit/hyperactivity disorder [10, 11]. Other signs and symptoms have been considered precursors of BD, including depressive symptoms, mood/ affective lability, hypomanic symptoms, and psychotic features [11–14]. Family history of BD is also considered a risk factor [15]. There is limited evidence about protective factors [16] although a healthy lifestyle, stable relationships, and social support seem to be beneficial [17].

Potential predictors for developing BD (“switching”) in this group include female sex, family history of mood disorders, psychotic features, emotional/behavioral dysregulation, hospitalization, and a younger age [9]. There is some evidence that individuals who develop BD from a depressive disorder may have differential characteristics and are considered “a special group” requiring further study [18].

A meta-analysis up to 2016 evaluated the risk of developing BD from major depressive disorder (MDD), including mostly adults [19]. Nearly a quarter of adults (22.5%) and adolescents with MDD followed up for a mean length of 12–18 years developed BD [19]. Another meta-analysis looking at conversion from unipolar depression to BD found that the rate of conversion to BD decreased with time from 3.9% in the first year after study entry with a diagnosis of unipolar depression to 3.1% in years 1–2, 1.0% in years 2–5 and 0.8% in years 5–10 [20]. Transition from MDD to BD was predicted by family history of BD (OR = 2.9), earlier age of onset of depression (*g* = −0.33), and presence of psychotic symptoms (OR = 4.76), based on 5–7 studies for each outcome [19].

In C&A, there is only one systematic review without meta-analysis, including only seven studies, looking at manic “switches” from MDD [21]. To our knowledge, no systematic review and meta-analysis has estimated the magnitude of the risk of developing BD from depressive disorders in C&A or carried out subgroup analyses or meta-regressions to detect predictive/moderating factors. Furthermore, while some of the evidence coming from adult meta-analysis may be relevant, it would require an update including the last 7–8 years as new studies have been published, for example, [9, 22–25]. Our aim was to estimate the risk of developing BD and the magnitude and consistency of moderating factors increasing the risk of developing BD in C&A with depressive disorders.

## Methods

This study was registered in PROSPERO (CRD42023431301). This systematic review and meta-analysis were conducted according to the PRISMA 2020 (eTable I) [26] and the MOOSE checklists (eTable II) [27], following the EQUATOR Reporting Guidelines [28].

### Literature search

A systematic search strategy was used to identify relevant articles, and a two-step literature search was implemented by independent researchers (G.S.P, V.P.R, J.E.d.O, and J.C). and Web of Science database (Clarivate Analytics) were searched from inception until July 1, 2023 (which then was updated on September 1, 2024). Web of Science database incorporates the Web of Science Core Collection, BIOSIS Citation Index, KCI-Korean Journal Database, MEDLINE, Russian Science Citation Index, and SciELO Citation Index as well as Cochrane Central Register of Reviews, and Ovid/PsychINFO databases. The following search terms were used in both searches: (“depres*” OR “depressive dis” OR “major depress* dis*” OR “major depression” OR” MDD”) AND (“adolesc*” OR “child*” OR “p?ediatric” OR “teen” OR “CAMHS” or “young people” OR “early-onset” OR “youth”) AND (“predict*” OR “onset” OR “risk of progression” OR “progression to mania” OR “transition” OR “threshold” OR “development” OR “switch”) AND (“mania” OR “manic” OR “bipolar disorder” OR “bipolar”).

Articles identified were first screened as abstracts, and after the exclusion of those which did not meet our inclusion criteria, the full texts of the remaining articles were assessed for eligibility and decisions were made regarding their inclusion in the review through consensus comparing Excel files. We completed our searches by manually reviewing the references of previously published articles and extracting any additional relevant titles. We used Google Scholar to look for these articles and particularly previous reviews. Discrepancies were resolved by consensus between the research team to reach a 100% agreement.

### Inclusion and exclusion criteria

Inclusion Criteria were: a) individual studies, b) conducted or providing stratified results on C&A (mean age < 18 years at baseline), c) with any depressive disorder diagnosis according to DSM/ICD criteria -any version-,) d) providing prospective longitudinal data on the development of DSM/ICD-defined BD (% of development/predictors of developing BD in C&A with depressive disorders), d) in any language. Exclusion criteria were: a) reviews, clinical cases, abstracts, conference proceedings, and study protocols, b) studies conducted in individuals with other designations, c) cross-sectional studies without information on the course of the illness, d) studies on remission, recurrence or relapse of BD only. Additional criteria for meta-analysis included independent/not overlapping studies at a certain follow-up point, and sample size being available for the analysis. This was determined by looking at the name of the program/cohort and the city/cities in which the study was conducted. Authors were contacted to clarify the % of development of BD and sample size when the exact figures were not clear.

### Data extraction

Three researchers (V.P.R, J.E.d.O, and J.C) independently extracted data from the included studies, using Microsoft Excel. Disagreements and doubts were solved by another author (GSP). Summary of included variables included the following information: first author and year of publication, country, age at baseline (mean age, SD, range), sex (% females), ethnicity, sample size, designation of patients (MDD, depressive disorders), diagnostic criteria (DSM, ICD), % who develop BD at follow-up, characterization (BD-I, BD-II, any BD which could include BD-NOS), treatments received (% antidepressants, % antipsychotics, % ADHD medication, % mood stabilizers), % family history of BD, % of hospitalization at baseline, % mood/affective lability as per established psychometric instruments, DSM-ICD defined comorbidity (% ADHD; % conduct disorders/ disruptive behavior disorders; % anxiety disorders, % substance use disorders), % subthreshold manic symptoms, age at onset of mood symptoms, follow up duration (in months) and quality assessment (see below).

### Risk of bias (quality) assessment

For study appraisal, we used the Newcastle–Ottawa Scale (NOS) [29]. This scale has three domains: selection, comparability, and outcome. The domain of selection has four categories assessing the representativeness of the sample, the sample size, the number of non-respondents, and the ascertainment of the exposure, with a maximum of five stars to be awarded. The domain of comparability has one category assessing if confounding factors are controlled for, with the maximum award of two stars. The final domain of outcome has two categories assessing the outcome and the appropriate usage of statistical tests, with the maximum award of three stars. Based on the number of stars in each category, the study quality may be assessed as good, fair, or poor.

### Strategy for data synthesis

First, the meta-analytical risk of developing BD, using the number of individuals with depressive disorders who developed BD was estimated as the primary outcome. Effect size was % with 95% CI. Note in none of the studies a prevalence of 0% was found so an adaptation for small counts or zero cells was not required.

Second, we meta-analyzed the % of individuals with depressive disorders who developed BD-I and BD-II separately. Third, we calculated the % BD-I and % BD-II within those groups, mainly for consistency. Fourth, we meta-analyzed separately the development of BD in studies with 1–3 years of follow-up, studies with 4–10 years of follow-up, and studies with >11 years of follow–up. Finally, we estimated the development of BD in samples of inpatient participants with depressive disorders.

Because the studies were expected to be heterogeneous, we used random-effects models. Heterogeneity among study point estimates was assessed with the Q statistic. The magnitude of heterogeneity was evaluated with the I^2^ index [30]. Publication bias was examined by visually inspecting funnel plots and applying the regression intercept of Egger [31], although note the limitations of publication bias assessments for proportion meta-analysis where both an increase and a decrease in the observed rates could lead to the decision of publishing or not a certain study [32].

Subgroup analyses were carried out by continent (Europe; Asia; North America; Others – South America, Australia), diagnostic classification (DSM – any version; ICD – any version), use of structured interviews, diagnosis (MDD only; combined depressive disorders), and study quality (good; fair; poor). Within-subgroup heterogeneity was calculated.

When the same variable was reported seven times or more [33, 34], meta-regressions evaluated the effect of the following variables on the development of BD: a) follow-up duration, b) % of females, c) mean age at baseline, d) % of hospitalization, e) % of family history of BD, f) % of anxiety disorders, g) % of white race, h) % of antidepressants, i) % of ADHD, j) % of conduct disorders, k) % substance use, l) % psychotic features, m) % recruitment from primary care, n) % recruitment from specialized services, and o) sample size Meta-regression β coefficients were calculated to test how the outcome variable changed with a unit increase in the meta-regression factors.

For the analysis, CMA version 3 was used [35]. All tests were 2-sided, and significance was set at *p* < 0.05.

## Results

### Sample characteristics

**Figure 1.**
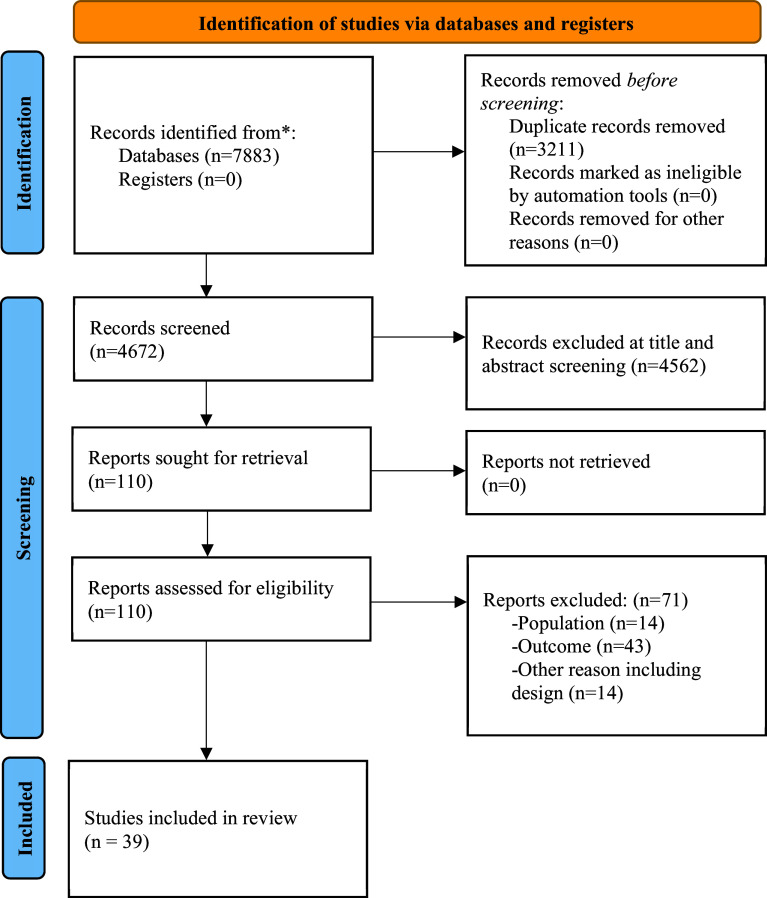
Preferred reporting items for systematic reviews and meta-analyses (PRISMA) flowchart outlining the study selection process.

The literature search yielded 4,672 citations, which were screened; 110 full-text articles were assessed for eligibility. After excluding those not meeting the inclusion criteria, 39 studies were included, reporting on 28 prospective independent cohorts (see Figure 1 for PRISMA flowchart).

The overall database, considering all independent samples, comprised 72,371 individuals with depressive disorders. The mean age of the participants at baseline was 13.9 years (mean age range 9.3–16.6 years), and 57.1% were females.

Most studies were carried out in North America (50.0%) and Europe (35.7%). The mean duration of the follow-up in these prospective cohorts was 112.8 months/9.4 years (range 1–25 years; eTable III).

### Risk of development of BD from a depressive disorder

**Figure 2.**
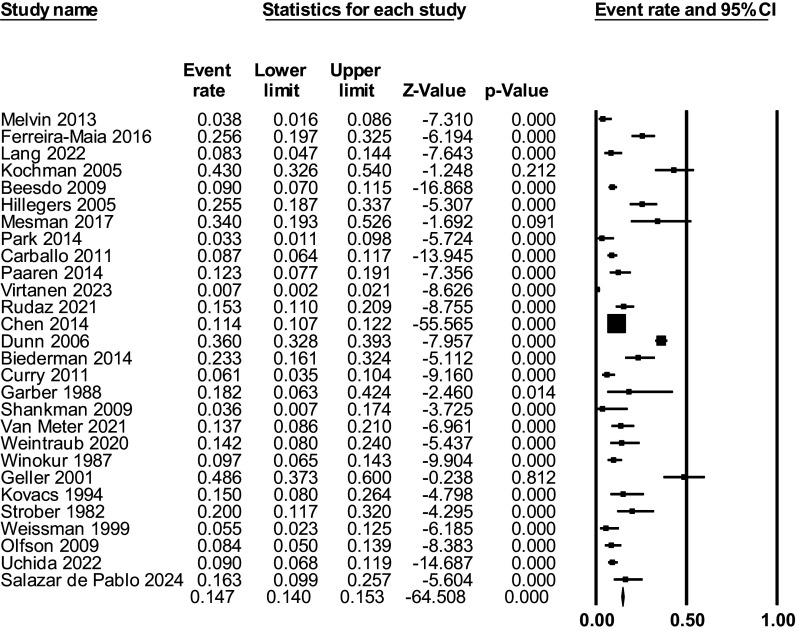
Development of BD in C&A with depressive disorders.

**Figure 3.**
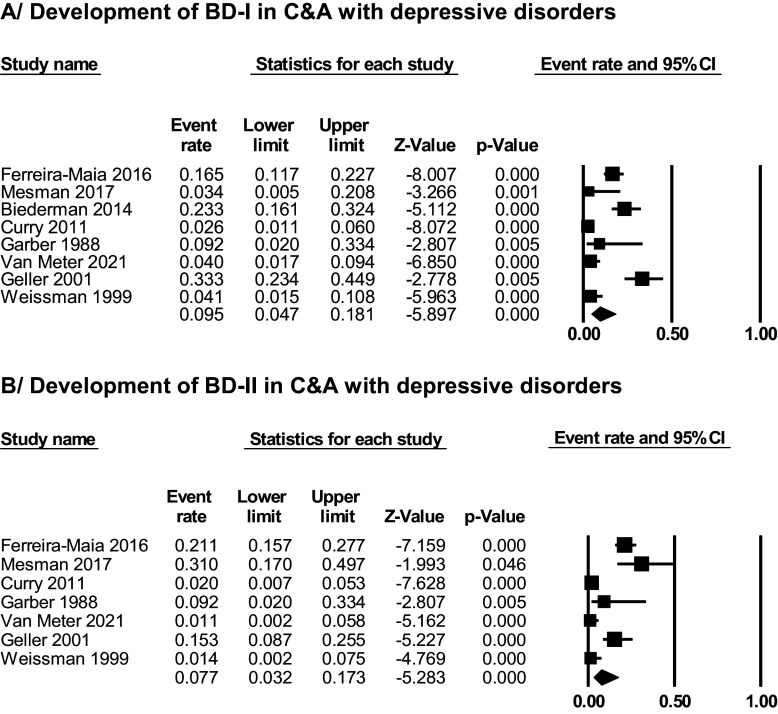
Development of BD-I and BD-II in C&A with depressive disorders.

In total, 14.7% of C&A with a depressive disorder developed BD at follow-up. Median follow-up was 102.8 weeks, interquartile range 52–155 weeks, and follow-up range from 20.4 to 288 months (K = 28; *N* = 72,371) (Figure 2). A subset of eight studies provided data for the development of BD-I or BD-II separately. In total, 9.5% of C&A with a depressive disorder developed BD-I (95% CI = 4.7% to 18.1%; K = 8; *N* = 2,045); 7.7% developed BD-II (95% CI = 3.2% to 17.3%) (K = 7, *N* = 1,942) (Figure 3). Within those who developed BD, meta-analytically 54.3% (95% CI = 38.1% to 69.6%) developed BD-I and 29.3% (95% CI = 16.4% to 46.9%) developed BD-II as the final diagnosis within the studied period.

In those studies, with a follow-up of 1–3 years, 15.7% (95% CI = 7.7% to 29.3%) of C&A with a depressive disorder developed BD (K = 7, *N* = 16,576); In those studies with a follow-up of 4–10 years, 10.1% (95% CI = 5.4% to 18.2%) of C&A with a depressive disorder developed BD (K = 11; *N* = 44,659). In those studies with a follow-up of >10 years, 14.0% (95% CI = 10.6% to 18.3%) developed BD (K = 11; *N* = 10,922).

Within C&A admitted into an inpatient unit with a depressive disorder, 19.8% (9.9% to 35.6%) developed BD. To note, excluding these studies the meta-analytical estimate for outpatient samples did not change (14.4, 95% CI 13.8% to 15.1% developed BD.

**Figure 4.**
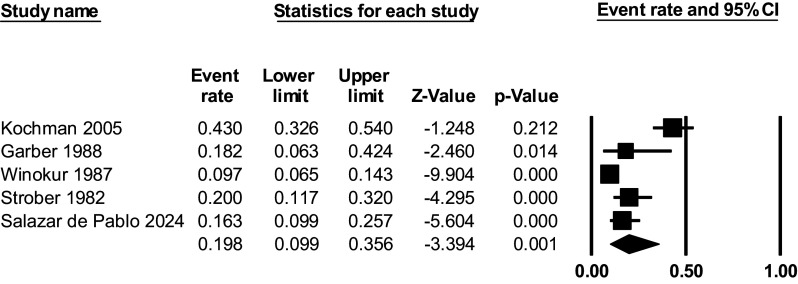
Development of BD in C&A with depressive disorders admitted into the hospital.

Median follow-up was 60 weeks, interquartile range 51.6–98.4 weeks, and follow-up range from 48 to 280 months (K = 5, *N* = 469) (Figure 4). Egger’s test (eTable IV) did not indicate the presence of publication bias (funnel plots available in eFigure I-III for the development of BD, BD-I, and BD-II). There was significant heterogeneity across studies, as indicated by high I^2^ (95.4%). Sensitivity analyses were conducted to explore potential sources of heterogeneity.

### Sub-group analyses and meta-regressions

Studies using the DSM to diagnose depressive disorders found higher rates of development of BD (21.6, 95% CI = 20.2% to 23.1%, K = 23, *N* = 3,607) than those using the ICD (11.1, 95% CI = 10.4% to 11.8%, K = 5, *N* = 68,764) (Q = 7.744, *p* = 0.005). Studies evaluating C&A with MDD found higher rates of development of BD (19.8, 95% CI = 16.8% to 23.1%, K = 9, *n* = 1,246) than those evaluating C&A with any depressive disorder (14.3, 95% CI = 13.6% to 15.0%, K = 19, *n* = 71,125) (Q = 13.515, *p* = 0.001).

Statistically significant differences according to the continent in which the study was conducted were not found (Q = 1.313, *p* = 0.726). In Europe, the development of BD occurred in 23.0% (95% CI = 21.3% to 24.8%) of C&A with depressive disorders (K = 10, *N* = 46,754). In Asia, the development of BD occurred in 11.3% (95% CI = 10.6% to 12.1%) of C&A with depressive disorders (K = 2, *N* = 7,360). In North America, the development of BD occurred in 13.7% (95% CI = 12.0% to 15.6%) of C&A with depressive disorders (K = 14, *N* = 17,940). In other continents (South America, Australia), the development of BD occurred in 20.6% (95% CI = 15.9% to 26.2%) of C&A with depressive disorders (K = 2, *N* = 317), although note the small sample size and heterogeneity. Differences according to the use of structured interviews (Q = 11.385, *p* = 0.001) were found to be the development of BD more common in those in which structured interviews were used (16.6, 95% CI = 12.0 to 22.6). Differences according to the quality of the studies were not found (Q = 2.254, *p* = 0.324). In “good” quality studies, the development of BD occurred in 15.1% (95% CI = 14.4% to 15.9%) of C&A with depressive disorders (K = 20, *N* = 26,962). In “fair” quality studies, the development of BD occurred in 12.1% (95% CI = 10.2% to 14.2%) of C&A with depressive disorders (K = 6, *N* = 1,507). In “poor” quality studies, the development of BD occurred in 7.0% (95% CI = 4.7% to 10.1%) of C&A with depressive disorders (K = 2, *N* = 43,902) (eTable V).

Younger age at baseline (β = −0.230, 95% CI = −0.436 to −0.025, *p* = 0.028), a history of hospitalization (β = 0.0162, 95% CI = 0.002 to 0.030 *p* = 0.025) and referrals from specialized clinics (β = 0.0121, 95% CI = 0.0044 to 0.199 *p* = 0.022) were associated with an increased risk of developing BD. Referrals from primary care (β = −0.011, 95% CI = −0.019 to −0.002 *p* = 0.0112) were associated with a decreased risk of developing BD. There was no association between the development of BD and other variables including the follow-up duration, % of females, % of individuals with family history of BD, % of individuals with anxiety disorders, % of individuals with white race, % of individuals taking antidepressants, % of individuals with comorbid ADHD, % of individuals with comorbid conduct disorders, % of individuals with substance use, % of individuals with psychotic features or sample size (*p* > 0.05; eTable VI).

### Quality of the included studies

The quality of the included studies was good in 30 studies (76.9%), fair in 7 studies (17.9%), and poor in 2 studies (5.1%), indicating overall good quality. Higher rating item was follow-up duration being enough (97.4% received a star). Lower rating item was selection of the non-exposed cohort as 38.4% did not provide a detailed description of the derivation of the cohort.

## Discussion

This is the first systematic review and meta-analysis to examine the risk of developing BD from depressive disorders and identify factors which moderate this development. We found that around 15% of C&A with depressive disorder developed BD and the risk increased to 20% for DSM-defined depressive disorders, MDD, and C&A requiring an inpatient admission into a psychiatric unit. Younger age at baseline and recruitment from specialized services were further associated with increased risk of developing BD. These findings highlight the substantial proportion of individuals with depressive disorders who develop BD, underscoring the clinical importance of early detection and intervention in this population.

Our findings demonstrate that the level of risk enrichment for developing BD in samples of C&A with depressive disorders is substantial, even during the first couple of years. To note, our meta-analytical prevalence was slightly lower, although comparable to the 22.5% of development of BD found in adolescents and young adults with MDD, followed up for a mean length of 12–18 years [19], that is, with longer follow-ups overall than the studies included in our review. This may be related to the focus on MDD and not other depressive disorders, and potentially the presence of a longer follow-up. However, it needs to be considered that between 23% and 55% of C&A with depressive disorders older than age 15 who convert to BD seem to do so within 1 year of being admitted for depression, and a further 36%–69% within 1–4 years of admission for depression [36]. Equally, the duration seems to be long enough to make early detection and intervention programs feasible [37]. In fact, in some cases, BD is developed after a decade or more [19]. A model of clinical trajectory of emergent BD in high-risk individuals defined transition stage as 1 when non-mood disorders appear; 2 when “minor” mood disorders such as depression NOS or dysthymia appear; 3 when major depressive disorder appears; and 4 when BD is developed [38]. This highlights the importance of depression as an “immediately” antecedent condition, and how BD may develop later on.

The association between hospitalization status and development of BD, as well as between MDD and BD compared to samples with other depressive disorders, suggests that individuals with more severe depressive symptoms may be at greater risk of developing BD. To note, there is some evidence suggesting that ICD could be more sensitive to detecting mild depressive disorders than the DSM which may detect more severe cases [39]. These findings would be concordant with our results showing that DSM-defined depressive disorders are also associated with an increased risk of developing BD. This finding has important implications for clinical practice, emphasizing the need for close monitoring and early intervention in individuals with severe depressive illnesses, both for BD and for other severe mental illnesses. A nationwide prospective 15-year register study on diagnostic conversion from unipolar depression to BD, schizophrenia, or schizoaffective disorder found a strong risk of developing these conditions in inpatient treatment settings. For late converters, the first “registration” of MDD during the teenage years was a further risk factor [22].

We found additional predictors which may play a role in the development of BD. An earlier age of onset seems to be associated with the development of BD in C&A with depressive disorders. Age at onset of MDD in individuals who did develop BD was 4.8 years (ES = 0.52) earlier than age at onset of MDD in those who did not develop BD [19]. This seems to be the case across mental health conditions, also for young people at clinical high risk for BD [40], and for recurrence of depression [41, 42]. To note, earlier onset is also associated with multiple indicators of greater illness burden across a wide range of indicators in MDD [43]. The positive association between younger age at baseline and increased development of BD underscores the importance of early intervention strategies targeting at-risk individuals with depressive disorders during childhood and adolescence.

Against our initial hypothesis, we did not find statistical evidence of an association between other factors such as antidepressant use and development of BD, although we may have lacked statistical power (only seven studies reported the % of C&A with depressive disorders on antidepressants). To note, a meta-analysis of 10 trials found that antidepressant treatment was associated with new mania-like responses in 8.2% of patients diagnosed with MDD [44], suggesting that manic symptoms need to be monitored in individuals with a depressive disorder who are started on antidepressants. Equally, we did not find evidence for an association between a positive family history of BD and development of BD. One hypothesis would be for the development of BD in C&A with a family history of BD to be linked to additional symptoms such as mood lability, or proximal hypomanic symptoms, as reported by previous studies [38, 45]. Risk calculators developed in this field have included dimensional measures of mania, depression, anxiety, and mood lability; but also psychosocial functioning and parental age at mood disorder as predictors for developing BD [46].

Our results have further clinical implications. A key consideration to avoid delays in establishing an adequate diagnosis would be to establish standard and accessible training packages and programs for mental health clinicians to detect BD and to evaluate symptoms of mania and hypomania, which are often under-recognized [47]. This detection would be particularly important in C&A with depressive disorders who continue to experience mental health difficulties. It is also important for clinicians to consider or be aware of alternative pharmacological options for young people at risk of BD (or who have developed BD). To note, lurasidone and the olanzapine-fluoxetine combination have been found to be efficacious for the treatment of bipolar depression in C&A [48], although note that the development of BD may be delayed until these individuals are adults. Evidence-based psychosocial interventions are also important to meet the needs of C&A who develop or who are at risk of developing BD [49]. For instance, interpersonal and social rhythm therapy (IPSRT) and specialist supportive care (SSC) used as an adjunct to pharmacotherapy appears to be effective in reducing depressive and manic symptoms and improving social functioning in adolescents and young adults with BD [50].

This study has some limitations that have to be taken into consideration. The most significant one is the heterogeneous and scattered findings across studies. To note, heterogeneity across studies may have influenced the overall estimates of BD development, and the associations observed in the meta-regression analyses. We tried to look for sources of heterogeneity by carrying out sub-group analyses and meta-regressions. Second, the variability in study methodologies and sample characteristics (note most studies came from Europe and North America) may limit the generalizability of the findings to other populations. However, with 39 studies and 72,371 individuals, it is reasonable to believe our sample is representative. Third, the sample size of several of the included studies was low. To note, in all the studies the sample size was enough to detect individuals who developed BD. Fourth, some studies used electronic health record data, which may be less accurate in identifying the development of BD. We did only consider studies categorizing individuals according to DSM or ICD criteria. Finally, we could not carry out meta-regression analyses on all the predictive factors we aimed to, including the % of antipsychotics, % of ADHD medication, % of mood/affective lability, % of alcohol use, % of melancholic features, % of atypical features, % of severe depression/suicidal ideation and % of subthreshold manic symptoms. The reason for this was an insufficient number of studies reporting this data and thus not having enough statistical power for these analyses in particular. Future research should focus on addressing these limitations by conducting large-scale prospective studies with standardized methodologies and rigorous assessment tools. Longitudinal studies with extended follow-up periods would also be recommended to further elucidate the trajectory of BD following a depressive disorder including the role of anti-depressant medication in the onset of BD, and identify potential protective factors that may mitigate or delay the risk of developing BD.

In conclusion, this study provides the first comprehensive estimate of the risk of developing BD from depressive disorders in C&A. Emerging evidence underscores the substantial risk of developing BD in C&A with depressive disorders, particularly those with MDD, DSM-defined depressive disorders, and C&A admitted into the hospital. The ability to accurately identify predictors of developing BD and understand the clinical trajectory of BD will allow clinician leaders to implement responsive BD care pathways in C&A mental health services. Provision of timely interventions will improve outcomes and reduce the burden of illness in affected individuals. Future research exploring additional predictors and preventive interventions is crucial for this purpose.

## Supporting information

Salazar de Pablo et al. supplementary material Salazar de Pablo et al. supplementary material

## Data Availability

Data were obtained from the cited articles.

## Supplementary material

The supplementary material for this article can be found at http://doi.org/10.1192/j.eurpsy.2024.1814.
