# Supplementary material for: Development and predictors of bipolar disorder in children and adolescents with depressive disorders: a systematic review, meta-analysis, and meta-regression
Source: Eur Psychiatry. 2025 Jan 8;68(1):e16. doi: 10.1192/j.eurpsy.2024.1814 (PMC11822963; doi:10.1192/j.eurpsy.2024.1814)
Supplement: Salazar de Pablo et al. supplementary material [file S0924933824018145sup001.docx]

**SUPPLEMENTARY MATERIAL**

**eTable I, page 2-4:** PRISMA statement and checklist

**eTable II, page 5-6:** Meta-analysis Of Observational Studies in Epidemiology (MOOSE) checklist

**eTable III, page 7-9:** Main characteristics of included studies

**eTable IV, page 10:** Egger’s test

**eTable V, page 11-12:** Sub-analyses and sensitivity analyses for development of BD

**eTable VI, page 13:** Meta-regressions relationship development of BD and moderating factors

**eFigure I, page 14:** Funnel Plot development of any BD

**eFigure II, page 15:** Funnel Plot development of BD-I

**eFigure III, page 16:** Funnel plot development of BD-II

**eFigure IV, page 17:** Meta-regression relationship age and development of BD

**eFigure V, page 18:** Meta-regression % of hospitalization and development of BD

**eFigure VI, page 19:** Meta-regression % recruitment from Primary Care and Specialized clinics

**This supplementary material has been provided by the authors to give readers additional information about their work.**

**eTable I: PRISMA statement and checklist**

| **Section and Topic** | **Item #** | **Checklist item** | **Location where item is reported** |
| --- | --- | --- | --- |
| **TITLE** | | |  |
| Title | 1 | Identify the report as a systematic review. | Title page |
| **ABSTRACT** | | |  |
| Abstract | 2 | See the PRISMA 2020 for Abstracts checklist. | Abstract page |
| **INTRODUCTION** | | |  |
| Rationale | 3 | Describe the rationale for the review in the context of existing knowledge. | Introduction |
| Objectives | 4 | Provide an explicit statement of the objective(s) or question(s) the review addresses. | Introduction |
| **METHODS** | | |  |
| Eligibility criteria | 5 | Specify the inclusion and exclusion criteria for the review and how studies were grouped for the syntheses. | Methods |
| Information sources | 6 | Specify all databases, registers, websites, organisations, reference lists and other sources searched or consulted to identify studies. Specify the date when each source was last searched or consulted. | Methods |
| Search strategy | 7 | Present the full search strategies for all databases, registers and websites, including any filters and limits used. | Methods |
| Selection process | 8 | Specify the methods used to decide whether a study met the inclusion criteria of the review, including how many reviewers screened each record and each report retrieved, whether they worked independently, and, if applicable, details of automation tools used in the process. | Methods |
| Data collection process | 9 | Specify the methods used to collect data from reports, including how many reviewers collected data from each report, whether they worked independently, any processes for obtaining or confirming data from study investigators, and if applicable, details of automation tools used in the process. | Methods |
| Data items | 10a | List and define all outcomes for which data were sought. Specify whether all results that were compatible with each outcome domain in each study were sought (e.g. for all measures, time points, analyses), and if not, the methods used to decide which results to collect. | Methods |
|  | 10b | List and define all other variables for which data were sought (e.g. participant and intervention characteristics, funding sources). Describe any assumptions made about any missing or unclear information. | Methods |
| Study risk of bias assessment | 11 | Specify the methods used to assess risk of bias in the included studies, including details of the tool(s) used, how many reviewers assessed each study and whether they worked independently, and if applicable, details of automation tools used in the process. | Methods |
| Effect measures | 12 | Specify for each outcome the effect measure(s) (e.g. risk ratio, mean difference) used in the synthesis or presentation of results. | Methods |
| Synthesis methods | 13a | Describe the processes used to decide which studies were eligible for each synthesis (e.g. tabulating the study intervention characteristics and comparing against the planned groups for each synthesis (item #5)). | Methods |
|  | 13b | Describe any methods required to prepare the data for presentation or synthesis, such as handling of missing summary statistics, or data conversions. | Methods |
|  | 13c | Describe any methods used to tabulate or visually display the results of individual studies and syntheses. | Methods |
|  | 13d | Describe any methods used to synthesise results and provide a rationale for the choice(s). If meta-analysis was performed, describe the model(s), method(s) to identify the presence and extent of statistical heterogeneity, and software package(s) used. | Methods |
|  | 13e | Describe any methods used to explore possible causes of heterogeneity among study results (e.g. subgroup analysis, meta-regression). | Methods |
|  | 13f | Describe any sensitivity analyses conducted to assess the robustness of the synthesised results. | Methods |
| Reporting bias assessment | 14 | Describe any methods used to assess risk of bias due to missing results in a synthesis (arising from reporting biases). | Methods |
| Certainty assessment | 15 | Describe any methods used to assess certainty (or confidence) in the body of evidence for an outcome. | Methods |
| **RESULTS** | | |  |
| Study selection | 16a | Describe the results of the search and selection process, from the number of records identified in the search to the number of studies included in the review, ideally using a flow diagram. | Result |
|  | 16b | Cite studies that might appear to meet the inclusion criteria, apart from those which were excluded, and explain why they were excluded. | Result |
| Study characteristics | 17 | Cite each included study and present its characteristics. | eTable III |
| Risk of bias in studies | 18 | Present assessments of risk of bias for each included study. | eTable III |
| Results of individual studies | 19 | For all outcomes, present, for each study: (a) summary statistics for each group (where appropriate) and (b) an effect estimate and its precision (e.g. confidence/credible interval), ideally using structured tables or plots. | Results, tables |
| Results of syntheses | 20a | For each synthesis, briefly summarise the characteristics and risk of bias among contributing studies. | Results |
|  | 20b | Present results of all statistical syntheses conducted. If meta-analysis was done, present for each the summary estimate and its precision (e.g. confidence/credible interval) and measures of statistical heterogeneity. If comparing groups, describe the direction of the effect. | Results |
|  | 20c | Present results of all investigations of possible causes of heterogeneity among study results. | Results, eTable IV-V |
|  | 20d | Present results of all sensitivity analyses conducted to assess the robustness of the synthesised results. | Results, eTable IV-V |
| Reporting biases | 21 | Present assessments of risk of bias due to missing results (arising from reporting biases) for each synthesis assessed. | Results |
| Certainty of evidence | 22 | Present assessments of certainty (or confidence) in the body of evidence for each outcome assessed. | Results |
| **DISCUSSION** | | |  |
| Discussion | 23a | Provide a general interpretation of the results in the context of other evidence. | Discussion |
|  | 23b | Discuss any limitations of the evidence included in the review. | Discussion |
|  | 23c | Discuss any limitations of the review processes used. | Discussion |
|  | 23d | Discuss the implications of the results for practice, policy, and future research. | Discussion |
| **OTHER INFORMATION** | | |  |
| Registration and protocol | 24a | Provide registration information for the review, including a register name and registration number, or state that the review was not registered. | Methods |
|  | 24b | Indicate where the review protocol can be accessed or state that a protocol was not prepared. | Methods |
|  | 24c | Describe and explain any amendments to the information provided at registration or in the protocol. | NA |
| Support | 25 | Describe sources of financial or non-financial support for the review and the role of the funders or sponsors in the review. | Discussion |
| Competing interests | 26 | Declare any competing interests of review authors. | Discussion |
| Availability of data, code and other materials | 27 | Report which of the following are publicly available and where they can be found: template data collection forms; data extracted from included studies; data used for all analyses; analytic code; any other materials used in the review. | Discussion |

**eTable II: Meta-analysis Of Observational Studies in Epidemiology (MOOSE) checklist** [1]

| **Criteria** | | **Brief description of how the criteria were handled in the meta-analysis** |
| --- | --- | --- |
| **Reporting of background should include** | |  |
| √ | Problem definition | No meta-analysis has evaluated the proportion of C&A with depressive disorder who developed BD. |
| √ | Hypothesis statement | We hypothesised that a significant proportion of C&A with depressive disorder would develop BD. |
| √ | Description of study outcomes | Developing BD would be the study outcome. |
| √ | Type of exposure or intervention used | Being diagnosed with a depressive disorder would be the study exposure. |
| √ | Type of study designs used | Cohorts. |
| √ | Study population | Subjects with a DSM/ICD-defined BD |
| **Reporting of search strategy should include** | |  |
| √ | Qualifications of searchers | Qualifications of researchers were specified.  The credentials of the investigators are indicated in the author list and in the acknowledgements. |
| √ | Search strategy, including time period included in the synthesis and keywords | We performed a multi-step literature search (keywords in the methods section) from inception until July 2023. |
| √ | Databases and registries searched | PubMed Web of Science database (Web of Science Core Collection, BIOSIS Citation Index, KCI-Korean Journal Database, MEDLINE, Russian Science Citation Index, and SciELO Citation Index). |
| √ | Use of hand searching | We hand-searched bibliographies of retrieved papers for additional references. |
| √ | List of citations located and those excluded, including justifications | Details of the literature search process can be found in the results section and PRISMA flowchart. |
| √ | Method of addressing articles published in languages other than English | Articles in any language were selected. We contacted native speakers to extract information in other languages. |
| √ | Method of handling abstracts and unpublished studies | Original individual studies; abstracts, and conference proceedings were included. Reviews were excluded. |
| √ | Description of any contact with authors | We contacted authors to gather additional data on the development of BD when not available in the article but there was a suggestion that the information may be available. |
| **Reporting of methods should include** | |  |
| √ | Description of relevance or appropriateness of studies.  to be tested | Detailed inclusion and exclusion criteria were described in the methods section. |
| √ | Rationale for the selection and coding of data | Data extracted from each of the studies were relevant to the population characteristics, study design, and the studies outcomes. |
| √ | Assessment of confounding | We conducted meta-analytical regressions whenever six or more studies were available to estimate the association between development of bipolar disorder and mediating factors. |
| √ | Assessment of study quality | We assessed the quality of the studies using NOS. |
| √ | Assessment of heterogeneity | Heterogeneity was assessed with the Q statistics. The proportion of the total variability in the effect size estimates was evaluated with the I^2^ index. |
| √ | Description of statistical methods in sufficient detail to be replicated | The effect size was estimated by calculating the proportion 95%CI. A random-effects meta-analysis was used. More details are described in the methods section. |
| √ | Provision of appropriate tables and graphics | We provided several tables and graphs in the main text and supplementary section to describe the literature search and its results. |
| **Reporting of results should include** | |  |
| √ | Table summarising individual study estimates and the overall estimate | We summarised individual study estimates and overall estimates in the text. |
| √ | Table giving descriptive information for each study included | We presented descriptive information for each study in the tables and as supplementary material. |
| √ | Results of sensitivity testing | Additional analyses were conducted as specified in the manuscript. |
| √ | Indication of statistical uncertainty of findings | We reported this in the results section. |
| **Reporting of discussion should include** | |  |
| √ | Quantitative assessment of bias | The presence of publication bias in the results was assessed informally by visually inspecting funnel plots. |
| √ | Justification for exclusion | We excluded studies based on the rationale of the meta-analysis as stated in the manuscript. |
| √ | Assessment of quality of included studies | The quality of the studies is discussed in the section. |
| **Reporting of conclusions should include** | |  |
| √ | Consideration of alternative explanations for observed results | Alternative explanations for observed results were considered. |
| √ | Generalisation of the conclusions | This point has been addressed in the discussion section. |
| √ | Guidelines for future research | Recommendations for future research were provided. |
| √ | Disclosure of funding source | This point has been addressed at the end of the manuscript. |

**eTable III**: **Main characteristics of included studies**

| **Author, year** | **Country** | **Mean age** | **% females** | **Ethnicity and race** | **Sample size: total; depressive disorders** | **Diagnosis: MDD vs depressive disorders; DSM vs ICD** | **Duration in months** | **QA** |
| --- | --- | --- | --- | --- | --- | --- | --- | --- |
| Baryshnikov 2020 [2] | Finland | (13-17) | 76.4 | N.a. | 6366; 6366 | Depressive disorders; ICD-10 | 180 | Fair |
| Beesdo 2020 [3] | Germany | (< 17) | N.a. | N.a. | 3021; 649 (Adults+adolescents) | Depressive disorders; DSM-IV | 120 | Fair |
| Biederman 2009 [4] | USA | 13.2, 3.6 (6–18) | 47.7 | N.a. | 522; 155 | Depressive disorders; DSM-III-R /KSADS-PL | 85.2 | Good |
| Biederman 2014 [5] | USA | (6-18) | 49.5 | N.a. | 522; 103 | Depressive disorders; DSM-III-R /KSADS-PL | 136.8 | Good |
| Carballo 2011 [6] | Spain | (13-17) | 60.3 | N.a. | 528; 443 | Depressive disorders; ICD-10 | 252 | Good |
| Chen 2015 [7] | Taiwan | N.a. | 59.6 | N.a. | 7270; 7270 | Depressive disorders; ICD-9-CM | 120 | Good |
| Curry 2014 [8] | USA | N.a. | 56 | 79% Caucasian, 9% Latinx, 8% African American, 4% Other | 196; 196 | MDD; DSM-IV | 60 | Fair |
| Dunn 2006 [9] | United Kingdom | 14, 1.6 (8-16) | 69.9 | N.a. | 113; 83 | Depressive disorders; DSM-III-IV | 93.6 | Good |
| Ferreira-Maia 2016 [10] | Brazil | 12.4, 3.7 | 48.2 | N.a. | 494; 349 | Depressive disorders; DSM-IV-TR | 20.4 | Good |
| Garber 1988 [11] | USA | 14.5 (10-17) | N.a. | 100% Caucasian | 20; 11 | Depressive disorders; DSM-III | 98.4 | Good |
| Geller 1993 [12] | USA | 10.1, 1.6 (6-12) | 40.7 | N.a. | 54; 54 | MDD; DSM-III | 36 | Good |
| Geller 1994 [13] | USA | 10.3, 1.6 (6-12) | 34.3 | N.a. | 110; 79 | MDD; DSM-III | 60 | Good |
| Geller 2001 [14] | USA | 10.3, 1.6 (6-12) | N.a. | N.a. | 72; 72 | MDD; DSM-III | 120 | Good |
| Jonsson 2011 [15] | Sweden | (16-17) | N.a. | N.a. | 382; 154 | MDD; DSM-III-R | 180 | Good |
| Kochman 2005 [16] | France | 12.7, 2.9 | N.a. | N.a. | 80; 80 | MDD; DSM-IV | 26.6 | Good |
| Kovacs 1994 [17] | USA | N.a. | N.a. | N.a. | 115; 60 | Depressive disorders; DSM-III | 88 | Good |
| Lang 2022 [18] | Finland | (13-17) | N.a. | N.a. | 55875; 1332 | Depressive disorders; ICD-10 | 336 | Good |
| Melvin 2013 [19] | Australia | 15.0, 1.5 | 66.3 | 98% White, 2% Asian | 140; 140 | Depressive disorders; DSM-IV | 108 | Good |
| Mesman 2017 [20] | Netherlands | 16 (12–21) | 50 | N.a. | 108; 29 | Depressive disorders; DSM-IV | 288 | Good |
| Musliner 2020 [21] | Denmark | (10-18) | 68.9 | N.a. | 7041; 7041 | Depressive disorders; ICD-10 | 120 | Good |
| Musliner 2018 [22] | Denmark | (10-19) | 63.2 | N.a. | 16724; 16724 | Depressive disorders; ICD-8, ICD-10 | 264 | Good |
| Olfson 2009 [23] | USA | N.a. | 54 | N.a. | 1274726; 16042 | Depressive disorders; ICD-9-CM | 24 | Good |
| Paaren 2014 [24] | Sweden | (16-17) | 84 | N.a. | 194; 130 | MDD; DSM-III-R | 180 | Good |
| Park 2014 [25] | South Korea | 14.7, 2.1 (8.8–18.3) | 57.7 | N.a. | 115; 90 | MDD; DSM-IV-TR | 10.2 | Good |
| Pfennig 2016 [26] | Germany | (14-24) | 61.6 | N.a. | 694; 694 | Depressive disorders; DSM-IV | 98.4 | Good |
| Reichart 2007 [27] | Netherlands | N.a. | N.a. | N.a. | 129; 31 | Depressive disorders; DSM-IV | 55 | Good |
| Rudez 2021 [28] | Switzerland | 10.4, 4.4 | 57.9 | N.a. | 449; 202 | MDD; DSM-IV | 167 | Good |
| Salazar de Pablo 2024 [29] | USA | 15.4±1.3 | N.a. | N.a. | 86; 86 | Depressive disorders; DSM | 60 | Good |
| Shankman 2009 [30] | USA | 16.6, 1.2 (14–20) | 59 | N.a. | 1709; 307 | MDD; DSM-III-R, DSM-IV | 300 | Good |
| Strober 1982 [31] | USA | 14.6 | 78 | N.a. | 60; 60 | Depressive disorders; DSM | 48 | Fair |
| Strober 1993 [32] | USA | 15.3 (3-18) | 64 | N.a. | 58; 58 | Depressive disorders; DSM | 24 | Fair |
| Uchida 2022 [33] | USA | (6-19) | 48 | N.a. | 492; 492 | Depressive disorders; DSM | 120 | Good |
| Van Meter 2021 [34] | USA | (6-12) | 29.8 | 68.1% White | 475; 121 | Depressive disorders; DSM-IV-TR | 96 | Good |
| Virtanen 2024 [35] | Sweden | (4-17) | 66.1 | N.a. | 43677; 43677 | Depressive disorders; ICD | 52 | Poor |
| Weintraub 2020 [36] | USA | 13.2, 2.6 | 64.6 | 81.9% White, 11.3% Hispanic, 6.8% other | 126; 75 | Depressive disorders; DSM-IV-5 | 25 | Fair |
| Weissman 1999a [37] | USA | 9.3, 1.9 (6-15) | 38.5 | 43.4% White, 28.9% Black, 26.5% Hispanic, 1.2% other | 300; 83 | MDD; DSM | 143 | Fair |
| Weissman 1999b [38] | USA | 14.7, 1.9 | 50.7 | 58.9% White, 9.6% Black, 27.1% Hispanic, 4.1% other | 134; 91 | MDD; DSM | 128 | Good |
| Winokur 1987 [39] | USA | N.a. | 55.1 | N.a. | 225; 225 | Depressive disorders; Pre-DSM (Feighner Criteria) | 51.6 | Poor |
| Zimmermann 2009 [40] | Germany | 14-24 | xc | N.a. | 2210; 488 | Depressive disorders; DSM-IV | 102 | Good |

DSM: Diagnostic and Statistical Manual of Mental Disorders; ICD: International Classification of Diseases; KSADS-PL: Kiddie Schedule for Affective Disorders and Schizophrenia - Present and Lifetime Version; MDD: Major Depressive Disorder; N.a.: Not available QA : Quality Assessment

**eTable IV: Egger’s test**

|  | **Intercept** | **SE** | **95%** | | **T value** | **P value** |
| --- | --- | --- | --- | --- | --- | --- |
| **Any BD** | **-0.003** | **1.274** | **-2.622** | **2.617** | **0.002** | **0.998** |
| **BD-I** | **-4.140** | **1.771** | **-8.475** | **0.194** | **2.337** | **0.058** |
| **BD-II** | **-3.670** | **1.493** | **-7.509** | **0.168** | **2.458** | **0.057** |

**eTable V: Sub-analyses and sensitivity analyses for development of BD**

| **Group**, subgroup | **No. of**  **Studies** | **Sample size** | **Effect size** | | | **z**  **Score** | **P** | **Test for Heterogeneity** | | | **Within subgroup heterogeneity** | |
| --- | --- | --- | --- | --- | --- | --- | --- | --- | --- | --- | --- | --- |
|  |  |  | **%** | **95 CI** | |  |  | **Q** | **I^2^** | **P** | **Q** | **P** |
| **Any continent** | **28** | **72371** | **14.7** | **14.0** | **15.3** | **-64.508** | **<0.001** | **592.466** | **95.443** | **<0.001** | 1.313 | 0.726 |
| Europe | 10 | 46754 | 23.0 | 21.3 | 24.8 | -23.668 | <0.001 | 278.307 | 96.766 | <0.001 |  |  |
| Asia | 2 | 7360 | 11.3 | 10.6 | 12.1 | -55.814 | <0.001 | 5.039 | 80.155 | 0.025 |  |  |
| North America | 14 | 17940 | 13.7 | 12.0 | 15.6 | -23.923 | <0.001 | 99.519 | 87.942 | <0.001 |  |  |
| Other (South America, Australia) | 2 | 317 | 20.6 | 15.9 | 26.2 | -8.426 | <0.001 | 20.818 | 95.197 | <0.001 |  |  |
| **Any diagnostic classification** | **28** | **72371** | **14.7** | **14.0** | **15.3** | **-64.508** | **<0.001** | **592.466** | **95.443** | **<0.001** | **7.744** | **0.005** |
| DSM | 23 | 3607 | 21.6 | 20.2 | 23.1 | -30.040 | <0.001 | 354.120 | 93.787 | <0.001 |  |  |
| ICD | 5 | 68764 | 11.1 | 10.4 | 11.8 | -58.775 | <0.001 | 30.452 | 86.865 | <0.001 |  |  |
| **All** | **28** | **72371** | **14.7** | **14.0** | **15.3** | **-64.508** | **<0.001** | **592.466** | **95.443** | **<0.001** | **11.385** | **0.001** |
| Use of structured interviews | 21 | 63714 | 16.6 | 12.0 | 22.6 | -8.238 | <0.001 | 322.862 | 93.805 | <0.001 |  |  |
| No use of structured interviews | 7 | 8657 | 7.2 | 5.0 | 10.3 | -12.757 | <0.001 | 35.624 | 83.156 | <0.001 |  |  |
| **Any diagnosis** | **28** | **72371** | **14.7** | **14.0** | **15.3** | **-64.508** | **<0.001** | **592.466** | **95.443** | **<0.001** | **13.515** | **0.001** |
| MDD only | 9 | 1246 | 19.8 | 16.8 | 23.1 | -13.840 | <0.001 | 109.650 | 93.616 | <0.001 |  |  |
| Depressive disorders | 19 | 71125 | 14.3 | 13.6 | 15.0 | -62.869 | <0.001 | 468.609 | 96.159 | <0.001 |  |  |
| **Any study quality** | **28** | **72371** | **14.7** | **14.0** | **15.3** | **-64.508** | **<0.001** | **592.466** | **95.443** | **<0.001** | 2.254 | 0.324 |
| Good | 20 | 26962 | 15.1 | 14.4 | 15.9 | -59.901 | <0.001 | 510.257 | 96.276 | <0.001 |  |  |
| Fair | 6 | 1507 | 12.1 | 10.2 | 14.2 | -21.052 | <0.001 | 39.707 | 87.408 | <0.001 |  |  |
| Poor | 2 | 43902 | 7.0 | 4.7 | 10.1 | -12.370 | <0.001 | 19.488 | 94.869 | <0.001 |  |  |

**Sensitivity analyses (probably not required)**

| **Group**, subgroup | **No. of**  **Studies** | **Sample size** | **Effect size** | | | **z**  **Score** | **P** | **Test for Heterogeneity** | | |
| --- | --- | --- | --- | --- | --- | --- | --- | --- | --- | --- |
|  |  |  | **%** | **95 CI** | |  |  | **Q** | **I^2^** | **P** |
| BD-I | 8 | 2045 | 9.5 | 4.7 | 18.1 | -5.897 | <0.001 | 60.093 | 88.351 | <0.001 |
| BD-II | 7 | 1942 | 7.7 | 3.2 | 17.3 | -5.283 | <0.001 | 46.786 | 46.786 | <0.001 |
|  |  |  |  |  |  |  |  |  |  |  |
| 1-3 y | 7 | 16576 | 15.7 | 7.7 | 29.3 | -4.10 | **<0.001** | 169.907 | 96.469 | **<0.001** |
| 4-10 | 11 | 44659 | 10.1 | 5.4 | 18.2 | -6.276 | **<0.001** | 1124.24 | 99.111 | **<0.001** |
| 11 | 11 | 10922 | 14.0 | 10.6 | 18.3 | -11.262 | **<0.001** | 135.793 | 92.636 | **<0.001** |
|  |  |  |  |  |  |  |  |  |  |  |
| % BD-I | 8 | 2045 | 54.3 | 38.1 | 69.6 | 0.510 | 0.610 | 99.705 | 92.530 | **<0.001** |
| % BD-II | 7 | 2045 | 29.3 | 16.4 | 46.9 | -2.287 | 0.022 | 77.824 | 92.290 | **<0.001** |

**eTable VI: Meta-regressions relationship development of BD and moderating factors**

|  | **N of studies** | **β Coefficient** | **SE** | **95% CI** | | **Z-Value** | **P value** |
| --- | --- | --- | --- | --- | --- | --- | --- |
| Follow-up duration | 28 | 0.0004 | 0.002 | -0.0036 | 0.0043 | 0.19 | 0.853 |
| % females | 13 | -0.003 | 0.031 | -0.063 | 0.057 | -0.09 | 0.926 |
| Mean age | 12 | -0.230 | 0.105 | -0.436 | -0.025 | -2.20 | **0.028** |
| % hospitalization | 9 | 0.0162 | 0.007 | 0.002 | 0.030 | 2.24 | **0.025** |
| % family history | 8 | 0.0049 | 0.006 | -0.006 | 0.016 | 0.85 | 0.397 |
| % anxiety disorders | 8 | 0.0011 | 0.023 | -0.045 | 0.047 | 0.05 | 0.964 |
| % white | 7 | -0.004 | 0.019 | -0.041 | 0.032 | -0.24 | 0.812 |
| % antidepressants | 7 | -0.027 | 0.022 | -0.070 | 0.016 | -1.23 | 0.219 |
| % ADHD | 7 | 0.015 | 0.017 | -0.018 | 0.049 | 0.88 | 0.376 |
| % conduct disorders | 7 | -0.027 | 0.044 | -0.113 | 0.059 | -0.62 | 0.537 |
| % substance use | 13 | 0.0316 | 0.022 | -0.0116 | 0.075 | 1.43 | 0.151 |
| % psychotic features | 8 | 0.0005 | 0.027 | -0.052 | 0.053 | 0.02 | 0.985 |
| % recruitment from primary care | 17 | -0.011 | 0.042 | -0.019 | -0.002 | -2.54 | 0.0112 |
| % recruitment from specialized clinic | 14 | 0.0121 | 0.040 | 0.0044 | 0.0199 | 3.07 | 0.022 |
| Sample size | 28 | -0.0000 | 0.001 | -0.0003 | 0.0002 | -0.26 | 0.794 |

**eFigure I: Funnel Plot development of any BD**

**eFigure II: Funnel Plot development of BD-I**

**eFigure III: Funnel plot development of BD-II**

**eFigure IV: Meta-regression relationship age and development of BD**

**eFigure V : Meta-regression % of hospitalization and development of BD**

**eFigure VI: Meta-regression % recruitment from Primary Care and Specialized clinics**

**REFERENCES**

[1] Stroup DF, Berlin JA, Morton SC, Olkin I, Williamson GD, Rennie D, et al. Meta-analysis of observational studies in epidemiology: a proposal for reporting. Meta-analysis Of Observational Studies in Epidemiology (MOOSE) group. JAMA. 2000;283(15):2008-12. <https://doi.org/10.1001/jama.283.15.2008>.

[2] Baryshnikov I, Sund R, Marttunen M, Svirskis T, Partonen T, Pirkola S, et al. Diagnostic conversion from unipolar depression to bipolar disorder, schizophrenia, or schizoaffective disorder: A nationwide prospective 15-year register study on 43 495 inpatients. Bipolar Disord. 2020;22(6):582-92. <https://doi.org/10.1111/bdi.12929>.

[3] Beesdo K, Höfler M, Leibenluft E, Lieb R, Bauer M, Pfennig A. Mood episodes and mood disorders: patterns of incidence and conversion in the first three decades of life. Bipolar Disord. 2009;11(6):637-49. <https://doi.org/10.1111/j.1399-5618.2009.00738.x>.

[4] Biederman J, Petty CR, Byrne D, Wong P, Wozniak J, Faraone SV. Risk for switch from unipolar to bipolar disorder in youth with ADHD: a long term prospective controlled study. J Affect Disord. 2009;119(1-3):16-21. <https://doi.org/10.1016/j.jad.2009.02.024>.

[5] Biederman J, Wozniak J, Tarko L, Serra G, Hernandez M, McDermott K, et al. Re-examining the risk for switch from unipolar to bipolar major depressive disorder in youth with ADHD: a long term prospective longitudinal controlled study. J Affect Disord. 2014;152-154:347-51. <https://doi.org/10.1016/j.jad.2013.09.036>.

[6] Carballo JJ, Muñoz-Lorenzo L, Blasco-Fontecilla H, Lopez-Castroman J, García-Nieto R, Dervic K, et al. Continuity of depressive disorders from childhood and adolescence to adulthood: a naturalistic study in community mental health centers. Prim Care Companion CNS Disord. 2011;13(5). <https://doi.org/10.4088/PCC.11m01150>.

[7] Chen MH, Chen YS, Hsu JW, Huang KL, Li CT, Lin WC, et al. Comorbidity of ADHD and subsequent bipolar disorder among adolescents and young adults with major depression: a nationwide longitudinal study. Bipolar Disord. 2015;17(3):315-22. <https://doi.org/10.1111/bdi.12266>.

[8] Curry JF. Future directions in research on psychotherapy for adolescent depression. J Clin Child Adolesc Psychol. 2014;43(3):510-26. <https://doi.org/10.1080/15374416.2014.904233>.

[9] Dunn V, Goodyer IM. Longitudinal investigation into childhood- and adolescence-onset depression: psychiatric outcome in early adulthood. Br J Psychiatry. 2006;188:216-22. <https://doi.org/10.1192/bjp.188.3.216>.

[10] Ferreira-Maia AP, Boronat AC, Boarati MA, Fu-I L, Wang YP. Evaluation of Bipolar Disorder in Children and Adolescents Referred to a Mood Service: Diagnostic Pathways and Manic Dimensions. J Psychiatr Pract. 2016;22(6):429-41. <https://doi.org/10.1097/PRA.0000000000000187>.

[11] Garber J, Kriss MR, Koch M, Lindholm L. Recurrent depression in adolescents: a follow-up study. J Am Acad Child Adolesc Psychiatry. 1988;27(1):49-54. <https://doi.org/10.1097/00004583-198801000-00008>.

[12] Geller B, Fox LW, Fletcher M. Effect of tricyclic antidepressants on switching to mania and on the onset of bipolarity in depressed 6- to 12-year-olds. J Am Acad Child Adolesc Psychiatry. 1993;32(1):43-50. <https://doi.org/10.1097/00004583-199301000-00007>.

[13] Geller B, Fox LW, Clark KA. Rate and predictors of prepubertal bipolarity during follow-up of 6- to 12-year-old depressed children. J Am Acad Child Adolesc Psychiatry. 1994;33(4):461-8. <https://doi.org/10.1097/00004583-199405000-00003>.

[14] Geller B, Zimerman B, Williams M, Bolhofner K, Craney JL. Bipolar disorder at prospective follow-up of adults who had prepubertal major depressive disorder. Am J Psychiatry. 2001;158(1):125-7. <https://doi.org/10.1176/appi.ajp.158.1.125>.

[15] Jonsson U, Bohman H, von Knorring L, Olsson G, Paaren A, von Knorring AL. Mental health outcome of long-term and episodic adolescent depression: 15-year follow-up of a community sample. J Affect Disord. 2011;130(3):395-404. <https://doi.org/10.1016/j.jad.2010.10.046>.

[16] Kochman FJ, Hantouche EG, Ferrari P, Lancrenon S, Bayart D, Akiskal HS. Cyclothymic temperament as a prospective predictor of bipolarity and suicidality in children and adolescents with major depressive disorder. J Affect Disord. 2005;85(1-2):181-9. <https://doi.org/10.1016/j.jad.2003.09.009>.

[17] Kovacs M, Akiskal HS, Gatsonis C, Parrone PL. Childhood-onset dysthymic disorder. Clinical features and prospective naturalistic outcome. Arch Gen Psychiatry. 1994;51(5):365-74. <https://doi.org/10.1001/archpsyc.1994.03950050025003>.

[18] Lång U, Ramsay H, Yates K, Veijola J, Gyllenberg D, Clarke MC, et al. Potential for prediction of psychosis and bipolar disorder in Child and Adolescent Mental Health Services: a longitudinal register study of all people born in Finland in 1987. World Psychiatry. 2022;21(3):436-43. <https://doi.org/10.1002/wps.21009>.

[19] Melvin GA, Dudley AL, Gordon MS, Ford S, Taffe J, Tonge BJ. What happens to depressed adolescents? A follow-up study into early adulthood. J Affect Disord. 2013;151(1):298-305. <https://doi.org/10.1016/j.jad.2013.06.012>.

[20] Mesman E, Nolen WA, Keijsers L, Hillegers MHJ. Baseline dimensional psychopathology and future mood disorder onset: findings from the Dutch Bipolar Offspring Study. Acta Psychiatr Scand. 2017;136(2):201-9. <https://doi.org/10.1111/acps.12739>.

[21] Musliner KL, Krebs MD, Albiñana C, Vilhjalmsson B, Agerbo E, Zandi PP, et al. Polygenic Risk and Progression to Bipolar or Psychotic Disorders Among Individuals Diagnosed With Unipolar Depression in Early Life. Am J Psychiatry. 2020;177(10):936-43. <https://doi.org/10.1176/appi.ajp.2020.19111195>.

[22] Musliner KL, Østergaard SD. Patterns and predictors of conversion to bipolar disorder in 91 587 individuals diagnosed with unipolar depression. Acta Psychiatr Scand. 2018;137(5):422-32. <https://doi.org/10.1111/acps.12869>.

[23] Olfson M, Crystal S, Gerhard T, Huang CS, Carlson GA. Mental health treatment received by youths in the year before and after a new diagnosis of bipolar disorder. Psychiatr Serv. 2009;60(8):1098-106. <https://doi.org/10.1176/ps.2009.60.8.1098>.

[24] Päären A, Bohman H, von Knorring L, Olsson G, von Knorring AL, Jonsson U. Early risk factors for adult bipolar disorder in adolescents with mood disorders: a 15-year follow-up of a community sample. BMC Psychiatry. 2014;14:363. <https://doi.org/10.1186/s12888-014-0363-z>.

[25] Park KJ, Shon S, Lee HJ, Joo Y, Youngstrom EA, Kim HW. Antidepressant-emergent mood switch in Korean adolescents with mood disorder. Clin Neuropharmacol. 2014;37(6):177-85. <https://doi.org/10.1097/WNF.0000000000000055>.

[26] Pfennig A, Ritter PS, Höfler M, Lieb R, Bauer M, Wittchen HU, et al. Symptom characteristics of depressive episodes prior to the onset of mania or hypomania. Acta Psychiatr Scand. 2016;133(3):196-204. <https://doi.org/10.1111/acps.12469>.

[27] Reichart CG, Wals M, Hillegers MH. [Children of parents with bipolar disorder]. Tijdschr Psychiatr. 2007;49(3):179-88.

[28] Rudaz D, Vandeleur CL, Gholam M, Castelao E, Strippoli MF, Marquet P, et al. Psychopathological precursors of the onset of mood disorders in offspring of parents with and without mood disorders: results of a 13-year prospective cohort high-risk study. J Child Psychol Psychiatry. 2021;62(4):404-13. <https://doi.org/10.1111/jcpp.13307>.

[29] Salazar de Pablo G, Frearson G, Radua J, Young A, Arango C, Kelleher I, et al. Cohort Study of Initially Hospitalized Adolescents with a Clinical Diagnosis of non-bipolar and non-psychotic mental disorders: frequency and correlates of development of bipolar-spectrum disorders. Under review. 2024.

[30] Shankman SA, Lewinsohn PM, Klein DN, Small JW, Seeley JR, Altman SE. Subthreshold conditions as precursors for full syndrome disorders: a 15-year longitudinal study of multiple diagnostic classes. J Child Psychol Psychiatry. 2009;50(12):1485-94. <https://doi.org/10.1111/j.1469-7610.2009.02117.x>.

[31] Strober M, Carlson G. Bipolar illness in adolescents with major depression: clinical, genetic, and psychopharmacologic predictors in a three- to four-year prospective follow-up investigation. Arch Gen Psychiatry. 1982;39(5):549-55. <https://doi.org/10.1001/archpsyc.1982.04290050029007>.

[32] Strober M, Lampert C, Schmidt S, Morrell W. The course of major depressive disorder in adolescents: I. Recovery and risk of manic switching in a follow-up of psychotic and nonpsychotic subtypes. J Am Acad Child Adolesc Psychiatry. 1993;32(1):34-42. <https://doi.org/10.1097/00004583-199301000-00006>.

[33] Uchida M, Bukhari Q, DiSalvo M, Green A, Serra G, Hutt Vater C, et al. Can machine learning identify childhood characteristics that predict future development of bipolar disorder a decade later? J Psychiatr Res. 2022;156:261-7. <https://doi.org/10.1016/j.jpsychires.2022.09.051>.

[34] Van Meter AR, Hafeman DM, Merranko J, Youngstrom EA, Birmaher BB, Fristad MA, et al. Generalizing the Prediction of Bipolar Disorder Onset Across High-Risk Populations. J Am Acad Child Adolesc Psychiatry. 2021;60(8):1010-9.e2. <https://doi.org/10.1016/j.jaac.2020.09.017>.

[35] Virtanen S, Lagerberg T, Takami Lageborn C, Kuja-Halkola R, Brikell I, Matthews AA, et al. Antidepressant Use and Risk of Manic Episodes in Children and Adolescents With Unipolar Depression. JAMA Psychiatry. 2024;81(1):25-33. <https://doi.org/10.1001/jamapsychiatry.2023.3555>.

[36] Weintraub MJ, Schneck CD, Walshaw PD, Chang KD, Sullivan AE, Singh MK, et al. Longitudinal trajectories of mood symptoms and global functioning in youth at high risk for bipolar disorder. J Affect Disord. 2020;277:394-401. <https://doi.org/10.1016/j.jad.2020.08.018>.

[37] Weissman MM, Wolk S, Wickramaratne P, Goldstein RB, Adams P, Greenwald S, et al. Children with prepubertal-onset major depressive disorder and anxiety grown up. Arch Gen Psychiatry. 1999;56(9):794-801. <https://doi.org/10.1001/archpsyc.56.9.794>.

[38] Weissman MM, Wolk S, Goldstein RB, Moreau D, Adams P, Greenwald S, et al. Depressed adolescents grown up. JAMA. 1999;281(18):1707-13. <https://doi.org/10.1001/jama.281.18.1707>.

[39] Winokur G, Wesner R. From unipolar depression to bipolar illness: 29 who changed. Acta Psychiatr Scand. 1987;76(1):59-63. <https://doi.org/10.1111/j.1600-0447.1987.tb02862.x>.

[40] Zimmermann P, Brückl T, Nocon A, Pfister H, Lieb R, Wittchen HU, et al. Heterogeneity of DSM-IV major depressive disorder as a consequence of subthreshold bipolarity. Arch Gen Psychiatry. 2009;66(12):1341-52. <https://doi.org/10.1001/archgenpsychiatry.2009.158>.
